# Trauma surgery workforce in the state of Rio Grande do Sul, Brazil

**DOI:** 10.1590/0100-6991e-20233495-en

**Published:** 2023-05-04

**Authors:** NEIVA BALDISSERA, MARIANA KUMAIRA FONSECA, CARLOS EDUARDO BASTIAN DA CUNHA, JOÃO ANTÔNIO MENEZES RIBEIRO, ROBERTA RIGO DALCIN, ROGÉRIO FETT SCHNEIDER

**Affiliations:** 1 - Hospital de Pronto Socorro de Porto Alegre, Serviço de Cirurgia Geral e do Trauma - Porto Alegre - RS - Brasil; 2 - Hospital Cristo Redentor/Grupo Hospitalar Conceição, Serviço de Cirurgia do Trauma - Porto Alegre - RS - Brasil; 3 - Hospital Pronto Socorro de Canoas, Serviço de Cirurgia Geral e do Trauma - Canoas - RS - Brasil

**Keywords:** General Surgery, Specialization, Surgical Procedures, Operative, Emergency Medical Services, Wounds and Injuries, Cirurgia Geral, Especialização, Procedimentos Cirúrgicos Operatórios, Serviços Médicos de Emergência, Ferimentos e Lesões

## Abstract

**Objective:**

to describe the current scope of certified trauma surgeons trained in the state of Rio Grande do Sul, including demographic data, geographic distribution, remuneration, and perspectives related to this specialty.

**Method:**

cross-sectional survey based on information collected through an electronic questionnaire sent to potential participants.

**Results:**

the response rate was 64% (n=75). There was a predominance of males (72%) with a mean age of 43 years. Most surgeons graduated from the Hospital de Pronto Socorro de Porto Alegre, and work in referral centers for trauma surgery in the capital and metropolitan region. More than 60% did not have any other training in a surgical subspecialty, though only a third stated that trauma surgery is their main source of income.

**Conclusion:**

trauma centers are poorly distributed and most surgeons work in referral hospitals in the metropolitan region of Porto Alegre. Due to the lack of recognition, limited financial income and shift work patterns, the career in trauma surgery care is unattractive, with only one third of surgeons performing most of their activities in this specialty.

## RESUMO

**Objetivo:**

descrever o perfil dos cirurgiões do trauma formados no estado do Rio Grande do Sul, incluindo dados demográficos, distribuição geográfica, remuneração, e perspectivas relacionadas à área de atuação.

**Método:**

estudo transversal do tipo inquérito, baseado em informações coletadas por meio de questionário enviado via plataforma digital para os profissionais da amostra em questão.

**Resultados:**

a taxa de resposta dos questionários foi de 64% (n=75). Houve predomínio do sexo masculino (72%) com idade média de 43 anos. A maior parte dos profissionais foi graduada pelo Hospital de Pronto Socorro de Porto Alegre, e atua em serviços de referência em Cirurgia do Trauma na capital e região metropolitana. Mais de 60% não realizou outra formação em subespecialidade cirúrgica, embora apenas um terço dos profissionais tenha declarado que a Cirurgia do Trauma seja sua principal fonte de renda.

**Conclusão:**

os centros de trauma são mal distribuídos e a maioria dos profissionais atua em hospitais de referência da região metropolitana de Porto Alegre. Devido ao baixo reconhecimento, incentivo financeiro limitado e desgaste da modalidade de trabalho em regime de plantão, a carreira com dedicação exclusiva na área de Cirurgia do Trauma é pouco atrativa, com apenas um terço dos profissionais desempenhando a maior parte de suas atividades na área.

## INTRODUCTION

Traumatic injuries have influenced the dynamics of mortality charts in recent decades, with a declining trend in deaths related to communicable diseases and an increase in deaths from external causes^1^. The World Health Organization (WHO) defines “external causes” as all deaths resulting from accidental and/or violent injuries, regardless of the time between their occurrence and the outcome. According to WHO data, trauma is responsible for claiming 4.4 million lives annually worldwide, representing 8% of all causes of death^2^. In Brazil, with an estimated 140,000 deaths/year, trauma is the third cause of death in the general population, and the first in the age group from 1 to 40 years^3^ ^-^ ^5^. Morbidity due to sequelae resulting from trauma is also the main cause of permanent disability in individuals younger than 45 years^1^ ^,^ ^3^ ^-^ ^5^. 

Trauma Surgery has General Surgery certification as prerequisite and take a leadership role in all phases in the care for victims of accidents and violence: initial assessment, diagnosis, surgical treatment, intensive care, follow-up during inpatient stay, and post-discharge follow-up. During specific training, the trauma surgeon is prepared to face all levels of this complex area, which requires, in addition to surgical technical skills, leadership capacity and training for immediate decision-making, confronting the imminence of death on a daily basis^6^ ^,^ ^7^.

Recognized by Resolution 1973/2011 of the Brazilian Federal Council of Medicine (CFM), the medical residency in Trauma Surgery exists since 1992, and the Hospital de Pronto Socorro de Porto Alegre (HPS) was the first service in the country to have a medical residency program (MRP) in this area. Currently, 25 MRPs in Trauma Surgery are recognized, distributed in nine states, offering 67 vacancies across the country^8^. The certification in Trauma Surgery can also be obtained through a selection held by the Brazilian College of Surgeons (CBC)^9^.

Despite its importance, trauma surgery is undervalued in our country. Currently, providing care for the third disease that kills the most in Brazil is not recognized as a distinct surgical specialty. Neither are there any determinations or regulations, by the authorities and health entities, that require hospitals to hire professionals with specific training in trauma care. In addition, Trauma Surgery has a limited number of vacancies in specific positions and public selection processes; it is difficulty to obtain accreditation for Trauma Centers; the wage is lower when compared with other surgical areas; there is a heavier workload and an on-duty work regime; and no career development plan. From this perspective, the present study proposes to evaluate the professional profile of trauma surgeons trained in MRPs in Rio Grande do Sul, in addition to their perceptions and perspectives regarding the present and future of this area of expertise.

## METHODS

This is a cross-sectional survey study, whose sample was defined by census, having as a reference population the trauma surgeons trained or working in MRPs in the state of Rio Grande do Sul - currently offered in reference hospitals in the capital of Rio Grande do Sul (Hospital de Pronto Socorro [HPS] and Cristo Redentor Hospital [HCR]) -, in addition to those certified by the CBC. There were no exclusion criteria. By the year 2021, 75 professionals had graduated from the HPS and 27 from the HCR, totaling 102 potential participants.

The data collection instrument consisted of a questionnaire prepared by the authors, containing 26 multiple-choice or simple selection items, which addressed topics such as training, perceptions, and perspectives of the participants about Trauma Surgery. The variables analyzed included sex, age, time elapsed since graduation and specialization, training hospital in Trauma Surgery, postgraduate degrees, teaching activities, subspecialty certification, practice in referral centers and/ or pre-hospital care, number of workplaces, practice in the public and/or private system, participation in residency preceptorship in Trauma Surgery, salary range, perceptions about the current MRP (duration, encouragement of young surgeons to pursue a career in trauma, and recognition of the area as a specialty), and participation in conferences and courses. The invitation to participate was announced through the communication channels of representative societies, and through direct and personal contact in message apps groups. Participation was conditioned to agreement with the Informed Consent Form.

The collected data were analyzed using the StatPlus software and described using frequency tables and position and dispersion measurements. To compare categorical variables, the Chi-square or Fisher’s exact tests were applied as appropriate, and comparisons between continuous variables were performed using the Student’s t test. Statistically significant associations were considered when p<0.05. The research protocol was submitted and approved by the Ethics in Research Committee of the Municipal Health Department of Porto Alegre, under the registration number 63001822.2.0000.5338, and the study report followed the recommendations of the Checklist for Reporting of Survey Studies (CROSS) of the EQUATOR Network initiative.

## RESULTS

The response rate of the electronic questionnaires was 64% (n=65). The sample was predominantly male (72%), with a mean age of 43 ± 11 years. The average number of years after training in Trauma Surgery was 13 ± 10 years, with graduations between 1994 and 2021. Both age and time elapsed since completion of medical residency showed a statistically significant difference between sexes. The mean age was 34 ± 7 in women and 46 ± 11 years among men (p<0.001), and as for years of practice, 6 ± 7 versus 16 ± 10, respectively (p<0.001).

The professionals were mostly graduated from the HPS (64%), and for 9% (n=6) of the participants there was no specific MRP at the time of their training. More than 60% of respondents (n=43) did not undergo training other than Trauma Surgery. Regarding their academic career, 63% (n=41) completed some level of stricto sensu postgraduate studies, although, of these, only 30% were trauma-related. Just over half (n=34) currently work in teaching. In this regard, we observed differences as to sex: 33% of women versus 60% of men (p=0.05) are professors.

Just over half of the professionals (n=50) work in referral trauma hospitals in the capital and metropolitan region, most of them at the Canoas Emergency Hospital, followed by the HCR and, finally, the HPS. Specialized care is also provided in private hospitals (n=10; 15%) and in the pre-hospital setting (n=8; 12%), to a lesser extent. Only nine (14%) did not work in the area after completing their residency and continue to work in hospitals that do not treat trauma patients. However, Trauma Surgery is the main activity of only one third of the participants (n=21) and the main source of income for only 21% (n=14).

At least 23% of participants work in more than one workplace. The proportion of women working in multiple jobs is higher (33% vs. 19%), though not statistically significant. Professionals who have Trauma Surgery as their main source of income work in more locations (median of 1.6 vs. 1.1 locations; p=0.009).

When asked about remuneration, one third of the surgeons (n=21) reported monthly income in the range of 20 to 30 thousand reais; however, the salary range showed great variability. For 65% (n=42), the remuneration of the trauma surgeon is not considered adequate. The majority (n=47, 72%) also mentioned the absence of salary bonuses according to professional qualification. Variation in salary range did not present a significant correlation with the number of workplaces or with age.

Only one participant does not believe that Trauma Surgery, which is now a recognized a subarea of General Surgery, should be considered a distinct medical specialty. The majority (n=56; 86%) defend that the residency should last for two years, instead of one. Regarding the recent change in the General Surgery MRP (which went from two to three years of specialization), 60% (n=37) believe that this will reduce the number of candidates for the MRP in Trauma Surgery.

As for training processes, continuing education, and participation in scientific activities, 74% (n=48) consider themselves updated in the area. Participation in national and international conferences on the subject is reported by 66% (n=43) and 28% (n=18) of professionals, respectively. Encouragement for research and scientific publishing in their workplaces, however, is cited by only 34% (n=22) of respondents. The sample profile is summarized in Table 1.

**Table 1:** Demographic data and sample profile. Data are reported in n (%) and mean ± standard deviation.

Sex (%)	
Female	18 (27.7)
Male	47 (72.3)
Age (years)	43 ± 11
Time since graduation (years)	18 ± 12
Time since specialization (years)	13 ± 10
Place of training in Trauma Surgery (%)	
Hospital de Pronto Socorro	42 (64.6)
Cristo Redentor Hospital	17 (26.2)
Certified by the Brazilian College of Surgeons	6 (9.2)
Postgraduation stricto sensu (%)	41 (63)
Training in subspecialty (%)	22 (33.8)
Number of workplaces (%)	
1	50 (76.9)
2	12 (18.5)
3	2 (3.1)
4	1 (1.5)
Teaching activity (%)	34 (52.3)
Preceptorship activity in Trauma Surgery (%)	18 (27.7)
Work at a trauma reference center (%)	
Pre-hospital	8 (12.3)
Hospital de Pronto Socorro	12 (18.5)
Cristo Redentor Hospital	16 (26.6)
Canoas Emergency Hospital	33 (50.8)
Trauma surgery as main source of income (%)	14 (21.5)
Monthly salary range (%)	
5-10 thousand reais	11 (16.9)
10-20 thousand reais	12 (18.5)
20-30 thousand reais	21 (32.3)
30-40 thousand reais	11 (16.9)
>40 thousand reais	10 (15.4)

**Figure 1:**
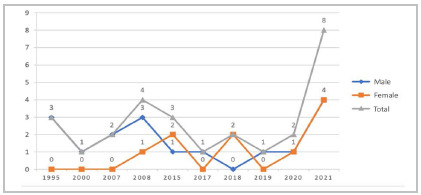
Evolution of the number of members of the Brazilian Society for Integral Care in Trauma (SBAIT), RS Chapter, over time, total and by sex.

Participation in the associative sphere within the Brazilian Society for Integral Care in Trauma (SBAIT) - RS Chapter has significantly increased in the last year, as shown in Figure 1. Currently, 27 trauma surgeons have active membership in the society.

## DISCUSSION

Surveys are commonly used to assess demographics and clinical practices of medical specialists. However, we did not find a similar study that sought to define the training and performance profile of trauma surgeons at the national, or even regional, level.

In the present study, 64% of potential respondents adhered, which is considered good. In three other surveys in the surgical area with a similar data collection methodology (via electronic forms), the percentage of responses ranged from 4.7 to 28% nationwide^10^ ^-^ ^12^. The electronic questionnaire is thus considered a good tool for data collection in research, and can be easily sent to the population under study through digital platforms, with the advantages of allowing the respondent to collaborate at an opportune moment, away from the presence of the researcher, and with guarantee of complete anonymity. We believe we obtained a good number of responses when compared to other studies because it was a regional study and most professionals worked in hospitals in the capital and metropolitan region, which facilitates the sending/resubmission of forms. 

Historically, the male sex has prevailed in medical practice in the country. A gradual increase in the female percentage in this area has been observed over the years and, according to the records of the Regional Councils of Medicine (CRM), women are already the majority among new doctors. Even with the growing female proportion in medicine, surgical specialties are still dominated by men^13^. We registered 28% of female trauma surgeons, similar to national and regional figures, which describe around 22% of female specialists in General Surgery^14^. 

This discrepancy is, in part, a reflection of the long period of patriarchal society, which attributed domestic/family roles and care to women, in addition to the stereotype of the surgical specialist as male^13^ ^-^ ^14^. It is believed that the trend that “medicine is increasingly feminine and young”, recorded by Scheffer et al.^13^, will also permeate surgical specialties. In our analysis, there was a statistically significant difference in the mean age between sexes, female surgeons being, on average, 12 years younger than male ones, which corroborates this trend.

**Figure 2:**
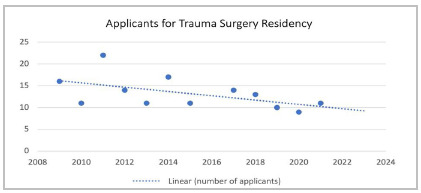
Number of applicants for the MRP in Trauma Surgery at the Porto Alegre Emergency. Linear trend dashed line.

Most respondents completed their training at the HPS in Porto Alegre, a pioneer in the creation of MRP in Trauma Surgery and the already recognized specialty of Emergency Medicine. The HPS has trained 75 trauma surgeons to date, and inspired the opening of other MRPs in the state and in Brazil. This service, considered a reference in the country, has observed a smaller number of applicants for MRP in Trauma Surgery in recent years (Figure 2). Similar cases have already been observed in other institutions in the country: recently, in the state of São Paulo, a MRP of surgical subspecialties registered a candidate/vacancy ratio of 30:1, 17:1, and 16:1 in Plastic Surgery, Colorectal Surgery, and Urology, respectively, while only 3:1 in Trauma Surgery^15^.

The increasing demand for certain surgical specialties is a growing phenomenon in the country and in the world. Recent graduates tend to prioritize quality of life and wages offered in the chosen specialty^16^ ^,^ ^17^. From this perspective, the on-duty shift work modality, characteristic of Trauma Surgery, is unattractive when evaluated in the long term, and there is no career plan that can compensate for this wear and tear over time.

For more than half of the respondents, the low demand for the certification in Trauma Surgery among young surgeons is due, at least in part, to the recent reformulation in the MRP of General Surgery, which included the third year for the conclusion of the specialty. This measure, nobly and assertively proposed by the CBC, increased training time from an undeniable perspective: the longer the training period, the better the skills acquired^18^. The new total workload for obtaining the title of General Surgeon, however, still remains lower than in other countries.

One would hope, under the same argument, that Trauma Surgery would also get an opportunity for the MRP’s reformulation agenda, which, in the perception of 86% of respondents, should be developed in a minimum period of two years. In fact, in August 2022, the Joint Committee of Specialties of CFM approved the request of the Brazilian Medical Association, changing the training time from one to two years^18^. However, this change may further reduce residents’ demand for Trauma Surgery MRPs.

In the present study, although three quarters of respondents work in referral hospitals and consider themselves up to date in the area, only 21% of professionals work exclusively with trauma and consider it their main source of income. We observed no difference in the average salary of these surgeons compared with others; however, the former work in more jobs. This corroborates the hypothesis that wages in Trauma Surgery are lower when compared with other surgical specialties, here covered by a greater workload. There are no reports in the literature about the average salary of trauma surgeons in other regions of the country.

**Figure 3:**
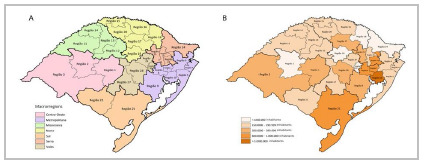
A) Health macro-regions of the State of Rio Grande do Sul; B) Population density of health macro-regions. Source: State Health Plan 2020-2023 of the State of Rio Grande do Sul.

The metropolitan region of Porto Alegre has three referral services for trauma patients. Although Trauma Care is foreseen in the Consolidation Ordinance GM/MS n. 03/2017, the Ordinance GM/MS n. 701/2018 extinguished the accreditation of new Trauma Centers, and these services, regrettably, are still not qualified as Trauma Centers in the state^19^. Together, the three institutions serve the entire metropolitan region and the Vales region (Figure 3), covering more than 152 cities that extend over an area of 57,197.75km^2^ , with a population of 5,997,880 people, more than half (52.7%) of the state’s population^19^.

The tendency for specialists to settle in the places where they completed their medical residencies is already reported in the literature and, in Rio Grande do Sul, it is certainly reinforced by the demand from the trauma referral centers in the region, which naturally absorb these professionals^16^ ^,^ ^20^. This distribution justifies the high percentage of respondents who work in the state’s referral services. On the other hand, it indicates that the other cities and regions of Rio Grande do Sul lack professionals with specific training. There is only one MRP vacancy in Trauma Surgery in the countryside, offered in the city of Santa Maria.

A study by a university in the interior of São Paulo that analyzed practices of the General Surgery MRP graduates describes that 77% work in surgical emergencies^17^. In this sample, only 4% had Trauma Surgery formal training. Similar results are described in other studies that analyze the emergency and urgent care of the Brazilian Public Health System (SUS), both clinical and surgical^16^ ^,^ ^20^. In this context, we point out a distressing reality: in the emergency wards, where training and experience are utmost for quick and assertive decisions, many newly graduated professionals are working without any qualification for caring for critically ill patients, and considering emergency shifts as a temporary job to supplement income until the end of the subspecialty^16^ ^,^ ^17^ ^,^ ^20^. 

A well-prepared trauma surgeon has the knowledge and skills to respond in the whole context that involves the trauma disease, also including prevention, study of the epidemiology and physics of trauma, prehospital care, and medical regulation. Trauma Surgery allows us to see life beyond the labels. Maybe this is truly the only place where we are all equal, regardless of sex, color, origin, social status, and background - noble or criminal. Everyone has the same probability of surviving, which increases when care is provided in the correct sequence, with the adequate infrastructure, and, mainly, by a surgeon sufficiently qualified to fully assist the complexity of the injuries. This “global surgical view” of trauma victims goes far beyond the basic training in General Surgery^7^ ^,^ ^21^ ^-^ ^27^.

The results presented here bring important reflections on the current and future generations of trauma surgeons. They also confirm the hypotheses already suggested and provide information for the development of strategies that encourage the valuing of Trauma Surgery training, thus bringing benefits for both patients and providers. Regrettably, the trauma disease has been neglected by the State, ignored by the population, and little valued by the medical community itself, which until now does not consider a specialist someone who bears in their title the same name as the disease - Trauma Surgeon.

As a limitation of the study, we highlight the small sample size, restricted to a single state of the Federation and which, despite the pioneering spirit in the creation of the specific MRP, currently has only seven vacancies/year for residents in Trauma Surgery. Therefore, it is not possible to extrapolate the results to other states or even to Brazil. Furthermore, non-probabilistic samples resulting from surveys carried out using self-completed electronic questionnaires may be a source of selection and response bias.

Finally, we highlight the role of SBAIT, which is the legitimate representative and main interlocutor in the fight against trauma in our country. As a scientific society, it actively participates in the improvement and training of the entire trauma team. Committed to the other surgical societies, all supported by basic training, without any dispute, conflict or competition, with the common and sworn interest of preserving life, SBAIT seeks recognition and appreciation of professionals who are truly capable of providing trauma care.

## CONCLUSION

Trauma is the leading cause of death and disability for young people and adults of working age. Despite the magnitude of the problem, specialized trauma care centers are poorly distributed in the state of Rio Grande do Sul, and trauma surgeons working in the region state that they have no recognition or any kind of incentive for dedication in this area. Compensation is lagged, forcing many professionals to work multiple jobs and look for alternative sources of income in General Surgery. In a society that is moving towards an era of recognized and well paid “super” specialists, this reality is undoubtedly not attractive for the young surgeon who is defining his future surgical career.
